# Decoding Melanoma Metastasis

**DOI:** 10.3390/cancers3010126

**Published:** 2010-12-30

**Authors:** William E. Damsky, Lara E. Rosenbaum, Marcus Bosenberg

**Affiliations:** 1 Department of Dermatology, Yale School of Medicine, New Haven, Connecticut, USA; E-Mails: William.Damsky@yale.edu (W.E.D.); Lara.Rosenbaum@yale.edu (L.E.R.); 2 Department of Pathology, University of Vermont College of Medicine, Burlington, Vermont, USA

**Keywords:** melanoma, metastasis, organ-specific, metastatic dormancy, pre-malignant dissemination

## Abstract

Metastasis accounts for the vast majority of morbidity and mortality associated with melanoma. Evidence suggests melanoma has a predilection for metastasis to particular organs. Experimental analyses have begun to shed light on the mechanisms regulating melanoma metastasis and organ specificity, but these analyses are complicated by observations of metastatic dormancy and dissemination of melanocytes that are not yet fully malignant. Additionally, tumor extrinsic factors in the microenvironment, both at the site of the primary tumor and the site of metastasis, play important roles in mediating the metastatic process. As metastasis research moves forward, paradigms explaining melanoma metastasis as a step-wise process must also reflect the temporal complexity and heterogeneity in progression of this disease. Genetic drivers of melanoma as well as extrinsic regulators of disease spread, particularly those that mediate metastasis to specific organs, must also be incorporated into newer models of melanoma metastasis.

## 1. Introduction

Melanoma is a major health problem and its rates are increasing both in the United States and worldwide. The estimated lifetime risk for development of melanoma is 1 in 74 compared with 1 in 1,500 in 1935 [1]. Melanoma has a predilection for metastasis early in disease progression, which can occur even from thin primary tumors [2]. Melanoma often has a protracted disease course, in which patients have a disease free period following surgical excision of the primary tumor only to discover visceral metastases months, years, or even decades later [3]. Melanoma metastasis is an ominous sign as it generally predicts poor prognosis. There are currently no FDA (U.S. Food and Drug Administration)-approved therapies which significantly improve overall survival for patients with late stage disease [4]. Much work has been done to understand mechanisms mediating the complex process of melanoma metastasis. Although substantial progress has been made in understanding these mechanisms, new data suggest this process is perhaps even more complicated than originally suspected.

Melanoma is a cancer that arises from melanocytes, which are the normal pigment-producing cells in the skin. Melanocytes are derived from the neural crest during development, to which they owe a complex and dynamic developmental history [5]. Melanocyte differentiation, survival, and migration from neural crest precursors rely heavily upon the canonical Wnt signaling pathway (through beta-catenin), the c-kit receptor tyrosine kinase, and downstream transcription factors such as MITF [6-8]. These and other pathways are frequently altered and may even be reactivated after transformation from melanocyte to melanoma [9-11]. In fact, reactivation of melanocyte-specific programs in the context of other oncogenic changes has been proposed to explain the proclivity of this tumor type to metastasize [12]. Notably, MITF, a transcription factor regulating melanocytic differentiation and pigment production, is known to be amplified in human melanomas, a finding that correlates with poor outcome [10].

**Figure 1.**
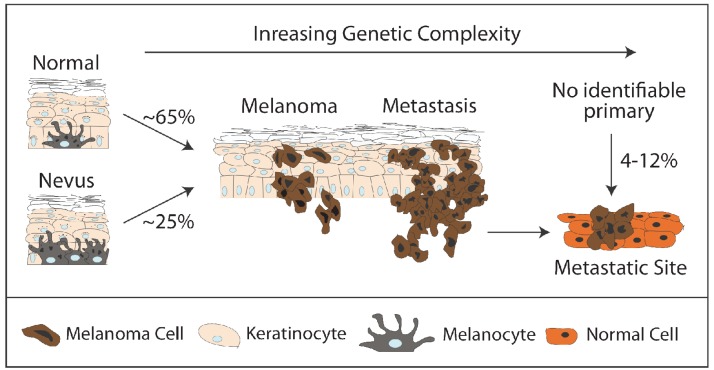
Origins of Metastatic Melanoma. Melanoma can arise either: (1) within a pre-existing melanocytic nevus (mole), generally associated with BRAFV600E mutation, or (2) with no visible precursor. Melanoma is thought to form within the epidermis, spread to the dermis, and ultimately to disseminate to distant sites (metastatic melanoma). Up to 12% of melanomas do not have an identifiable cutaneous precursor lesion. Progression of any individual lesion is thought to be driven by the acquisition of additional genetic/epigenetic changes.

Though the majority of melanocytes are found within the skin, normal melanocytes can also be found in other anatomic locations including the uvea of the eye. In normal adult skin, melanocytes are found at the junction of the dermis and epidermis, just superficial to the basement membrane (Figure 1). Developmentally, melanocytes must cross the basement membrane in order to reach this position [13] and may retain a predisposition for this ability. Melanocytes exist primarily as individual cells within the epidermis, and rather than forming homotypic interactions with each other, form heterotypic interactions with neighboring keratinocytes, the predominant cell type in skin. Melanocytes within the epidermis adopt some epithelial features, including the formation of adherens junctions to neighboring keratinocytes [14], but are not epithelial cells themselves. Normal melanocytes produce and subsequently transfer pigment-producing melanosomes to neighboring keratinocytes, which is thought to protect the keratinocytes from the damaging effects of UV (ultraviolet) radiation.

## 2. Melanoma Formation and Progression

Progression from normal melanocytes to melanoma has classically been divided into a series of progressive steps [15]. Although there are several histologic subtypes of melanoma, this model best describes superficial spreading melanoma, the most common variant, but is useful in understanding other subtypes as well. Melanoma is thought to arise in one of two ways: (1) with no visible precursor lesion or (2) in association with a benign melanocytic proliferation called a nevus (or mole). Although only 20–30% of melanomas are thought to arise in association with a nevus precursor [16-18], this model is also useful in understanding the progression of *de novo* melanoma (Figure 1).

In the progression model of melanoma, the first step is the formation of a nevus, which is a proliferation and aggregation of melanocytes into nests located at the epidermal/dermal junction or within the dermis. In some nevi, cytological atypia and an altered growth pattern are present, which have been defined as features of a dysplastic nevus [15]. The next step is progression from nevus to melanoma *in situ.* Melanomas that do not develop from a precursor lesion may be first detected in this stage. Melanoma *in situ* is confined to the junction of epidermis and dermis. In the next step of the progression model, invasive malignant melanoma cells grow into the dermis where they interact with many new cell types and gain physical access to both lymphatics and blood vessels. The final step of progression is to metastatic melanoma, where tumor cells have spread from the primary site and established foci of disease at distant sites. It has been proposed that the progression through these steps is associated with the accumulation of genetic and epigenetic changes, a subset of which are thought to drive the process forward and provide melanocytes with increasingly malignant potential [19].

Melanoma is often initiated by the formation of a benign, growth-arrested nevus. As such, the genetic changes of these nevi have been a focus of research. About 80% of human melanocytic nevi contain an activating mutation (V600E) in the BRAF Ser/Thr kinase [20,21]. BRAF is a regulator of the MAPK/ERK pathway, which positively regulates cell cycle progression. This oncogenic change, which makes BRAF constitutively active, is thought to drive the initial melanocytic proliferation that forms the nevus. Although the mechanisms regulating subsequent growth arrest are poorly understood, various mechanisms have been proposed to mediate this process and are termed oncogene-induced senescence [22]. The prevalence of BRAFV600E mutations in melanocytic nevi suggests that BRAF mutations may be important initiating events, but do not inexorably lead to melanoma, as the estimated lifetime risk of progression of a particular nevus to melanoma is roughly 1 in 7,000 [23].

Although the model described above provides a foundation for understanding melanoma formation and progression, the critical events that occur between local tumor expansion and metastatic spread are complex and not addressed by the model. Additionally, there is compelling evidence that progression does not always occur in such a neat, step-wise fashion. In fact, there is evidence to support the notion that melanocytic cells can spread to distant sites in earlier stages of tumor progression. Lastly, melanomas show a predilection for metastasis to particular organs. Much work has been done to explain these phenomena at a molecular level and these issues will be the focus of this review.

## 3. Tumor Heterogeneity and the Metastatic Cascade

Melanoma initiation is thought to be a clonal event [24], but tumors subsequently evolve and acquire heterogeneity owing to selective pressures within the tumor microenvironment and the acquisition of genomic instability [25]. Within such heterogeneity, it has classically been thought that relatively rare populations of cells gain the ability to spread and only metastasize very late in tumor progression [26]. These rare tumor cells are thought to drive the metastatic process, which is also thought to be a clonal event [27,28]. At the distant metastatic site, tumor cells have been proposed to continue a period of uninterrupted growth, resulting in clinically evident disease. Heterogeneity also develops in the metastasis due to selective pressures, and these metastases may in turn seed additional metastases to other sites [29].

The relationship between tumor heterogeneity and metastasis was an early focus of cancer and metastasis research. In the early 1970s, using the B16 mouse model of melanoma, Isiah Fidler showed that melanoma cells had both heritable and selectable phenotypic traits that influenced their ability to metastasize [30]. This work provided early experimental evidence that certain cells within the same primary tumor may have an enhanced ability to metastasize. Experimental proof for this hypothesis came four years later [31]. In these studies, different clones derived from the same parent melanoma cell line were shown to have differing abilities to form lung metastasis after intravenous injection into mice. Some years later, metastatic heterogeneity in individual human melanomas was experimentally confirmed using similar experiments in which clones derived from human melanoma cell lines were injected into nude mice [32].

**Figure 2.**
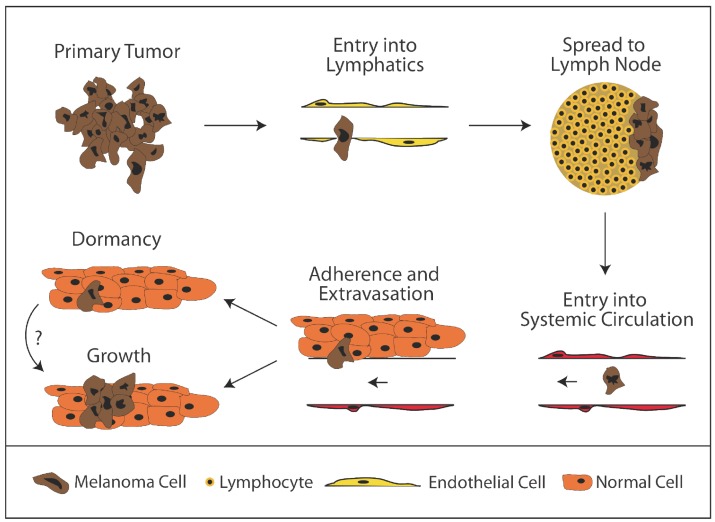
Steps in Melanoma Metastasis. After formation of a primary tumor, melanoma cells are thought to enter into lymphatic vessels, traverse to the lymph node, and subsequently enter into systemic circulation via the thoracic duct. After reaching systemic circulation, cells must adhere to the microvasculature of a target organ, extravasate, and subsequently proliferate in order to form a clinically relevant metastasis. The mechanisms regulating either success or failure at any step are likely important and probably differ amongst different melanomas and different target organs.

Given that metastasis is a complex process, it is not surprising that individual tumor cells may be better than others in carrying out this process. In order for a tumor cell to metastasize and form a clinically detectable and potentially lethal metastasis, it must complete a series of steps (Figure 2). After primary tumor formation, tumor cells must gain access to systemic circulation in order to spread to distant sites. In melanoma, this is thought to occur primarily by entry of tumor cells into a lymphatic vessel, transit through a lymph node, and entry into systemic circulation via the thoracic duct [33]. Once in circulation, the tumor cells must not only survive but must also adhere to an endothelium within a target organ. After adhering, tumor cells must extravasate into the parenchyma of the target organs. Here, the tumor cells find themselves in foreign microenvironments in which they must survive. If they survive, in order for clinically detectable disease to form, the cells must find a way to proliferate. In many cancers, including melanoma, clinically apparent metastases are primarily found only within a subset of organs, suggesting that something inherent to different organs may facilitate growth at these sites.

In recent years, many studies have focused on the processes regulating the ability of tumor cells to enter circulation, a process that is thought to be related to their ability to invade normal surrounding tissue [34]. However, as can be seen, this is only one step of a long, complex process. There is evidence to suggest that other steps of the process, such as those regulating the ability of metastatic tumor cells to adhere, extravasate, survive, and grow in the target organ, may be equally or more important. For example, the presence of circulating tumor cells is common and does not necessarily predict metastasis accurately [35,36]. Factors regulating these processes in target organs may represent the rate-limiting step in many metastases.

## 3. The Seed-and-Soil Hypothesis

The patterns of metastasis observed in human malignancy have long been of research interest. Early studies by Rudolf Virchow, and later James Ewing, proposed that the patterns of metastasis observed in human malignancy might be explained by the anatomy of human circulation [37,38]. In other words, tumor cells that embolized from the primary tumor and gained access to systemic circulation would arrest and grow indiscriminately in any tissue in which they happened to find themselves. However, in 1889, Stephen Paget presented an alternative theory to explain the patterns of metastasis observed in human malignancy [39]. Paget proposed that the anatomy of circulation alone could not account for the metastatic patterns that he observed in a series of autopsies on breast cancer patients. Instead, he suggested that factors inherent to certain tumor types (the seeds) may allow them to preferentially grow in certain organs (the soil). This hypothesis, often referred to as “seed-and-soil,” has largely been supported by more than a century of experimental testing.

Many years after Paget's seed-and-soil hypothesis was proposed, Ian Hart and Isiah Fidler provided experimental confirmation of this hypothesis using the B16 mouse model of melanoma [40]. After intravenous injection of melanoma cells, they observed that tumor cells preferentially adhered to experimental pulmonary grafts, but not to control renal grafts. Interestingly, this set of experiments used radioactive tracing of tumor cells to show that rates of initial arrest were similar in both organs, but growth of metastases was different. These results provided early evidence that the ability of tumor cells to interact with, survive in, and proliferate at distant sites are important aspects of metastasis formation.

Compelling evidence for the seed-and-soil hypothesis in humans came from a study in ovarian cancer patients. Ovarian cancer is known to primarily spread within the abdominal cavity and in fact >90% of all ovarian cancer metastases are thought to be confined to the peritoneum [41]. One hypothesis to explain these findings is that ovarian cancer cells do not enter systemic circulation, and thus have no opportunity to grow at distant sites. An alternative hypothesis suggests that ovarian cancer cells do actually enter systemic circulation, but prefer to grow in the “soil” of the peritoneal cavity. In 1984, Tarin and colleagues provided a direct test of this hypothesis in human ovarian cancer patients. This study followed a group of ovarian cancer patients with ascites in which peritovenous shunts were placed to drain ascitic fluid. Not only did these shunts provide therapeutic relief of ascites, they also provided tumor cells with direct access to the systemic circulation. Remarkably, the presence of these peritovenous shunts did not increase metastasis outside of the peritoneal cavity. This incidental, but resourceful, analysis provides strong *in vivo* support for the seed-and-soil hypothesis in humans.

In fact, compelling evidence for the seed-and-soil hypothesis is quite common in the literature. Two such examples are prostate cancer and ocular melanoma. Prostate cancer is known to metastasize primarily to bone and only infrequently to other sites [41,42]. Ocular melanoma, which is perhaps one of the most intriguing examples in all of cancer, primarily metastasizes from the eye to the liver, with 87% of metastatic uveal melanoma patients exhibiting liver metastasis [43]. In fact, fluorescently-tagged uveal melanoma cells injected intravenously into nude mice persist only in the liver [44]. Uveal melanoma also exemplifies a process known as metastatic dormancy (see Section 6 below: Dormancy in Metastatic Melanoma) in which growth-arrested, disseminated tumor cells can persist for years to decades, only to resume growth later as clinically detectable metastases [26].

In many types of cancer, there is compelling evidence for the seed-and-soil hypothesis, however in some cases the anatomy of vascular tumor drainage plays a central role. For example, in colorectal carcinoma, 80% of patients with metastases show metastasis to the liver, a pattern proposed to be governed by the course of the mesenteric circulation to and through the liver [41]. There are also hints that anatomy may influence melanoma metastasis. For example, primary melanoma located on the trunk, head, and neck are more likely to recur than those located in the extremities [45], though there are certainly other potential explanations for this observation.

## 5. Clinical Considerations in Melanoma Metastasis

Improvements in awareness have increased detection of melanoma, such that many melanomas are diagnosed early in disease progression [1]. Though surgical excision is generally thought to be curative in these patients, a subset will develop recurrent disease. While melanomas rarely recur locally at the site of excision, they often recur as metastases at distant sites [46]. Even in Stage IA melanoma patients, who have a 20-year survival rate of at least 90%, recurrences of disease still occur, often a decade or more after the removal of the primary tumor [47].

Melanoma metastasis cause the vast majority of morbidity and mortality associated with this disease. The presence of metastasis to visceral sites predicts poor outcome in melanoma [3]. The one-year survival rates in melanoma patients with clinically apparent metastasis to one, two, or three different visceral sites is: 36%, 13%, and 1%, respectively [48]. The most important tumor intrinsic variable that can predict metastatic recurrence in early melanomas is the thickness of the tumor [49,50]. Prognosis is inversely proportional to tumor thickness. Strikingly, differences of only 1–2 mm in the thickness can alter prognosis substantially [3].

Sentinel lymph node dissection is generally offered to patients with melanomas exceeding 1 mm in depth. The sentinel node is the first lymph node encountered by fluid draining from the cutaneous site of the primary tumor and is thought to represent the first non-contiguous site tumor cells will spread to [51]. Although removal of the sentinel node, or even the entire nodal basin does not improve longterm survival [52,53], the presence or absence of tumor cells in the sentinel node has very important prognostic implications [3]. In fact, histological evidence of tumor cells in the lymph node is probably the best indication that sub-clinical spread of melanoma cells has already occurred.

In addition to primary tumor thickness and sentinel lymph node status, other factors have been correlated with the probability of metastatic recurrence and some of these factors have been incorporated into the most recent melanoma staging system [47]. For example, the odds of metastasis from thin lesions are three-fold higher in men [54]. The age of the patient and anatomic location can also influence recurrence rates [50,54]. Factors such as the presence of ulceration, microscopic satellite lesions, and increased mitoses per high power field all predict poor outcome [54]. The presence or absence of tumor infiltrating lymphocytes can also be correlated with outcome [54] though the relationship between inflammation and melanoma is quite complex and will not be reviewed in detail here.

Melanomas are capable of metastasizing to both regional and distant sites. The most common sites of regional metastasis are nearby skin, sub-cutaneous tissue, and lymph nodes [48]. Metastases to skin are referred to as either satellite lesions (if they are relatively close to the primary tumor) or in transit metastases (if they are relatively more distant), though do not differentially influence melanoma staging [47]. Metastasis to the skin may be the first external clue that lymphatic or hematogenous spread has occurred [55].

The most common clinically apparent sites of distant metastases in melanoma patients are: skin, lung, brain, liver, bone, and intestine [48]. Metastasis to lung is common and often the first clinically apparent site of visceral metastasis. Other sites of metastasis such as bone and intestines occur later in disease progression and are rarely the first site of detection [48]. Metastases to other sites such as liver and brain are inversely correlated to each other [48]. In an autopsy series of melanoma patients, it appears that metastatic tumor burden, especially in terminal patients, is actually much higher than is clinically appreciated. A generally higher preponderance of metastases to sites frequently encountered clinically is observed at autopsy. For example, intestinal metastasis is detected in only 1–7% of patients in clinical series, but 26–58% of patients at autopsy [48]. In analysis of autopsy series it is also evident that subclinical melanoma metastases can manifest in almost any part of the body [48,56]. These types of metastases are not infrequently found in: the heart, pancreas, adrenal glands, spleen, stomach, esophagus, thyroid gland, kidneys, genitals, blood vessels, and more [48,56]. In fact, it seems there are few places that melanoma is not capable of metastasizing to, especially in late stage disease. Understanding why melanoma becomes clinically apparent in certain organs and not others is of much interest in organ-specific metastasis research. Lack of detection in some sites may be due to technical issues related to imaging, but certainly also represents differences in biological interactions between tumor cells and different organs.

## 6. Dormancy in Metastatic Melanoma

The time period between removal of the primary tumor and subsequent recurrence of disease is referred to as metastatic dormancy. In melanomas, a period of dormancy may end with the emergence of recurrent disease at a metastatic site and only rarely at the site of the primary tumor. Melanomas, as well as some other cancers, such as prostate and some types of breast cancer, often have very protracted courses in which metastatic disease does not manifest until years or even decades after removal of the primary tumor. Clinically localized melanoma can recur after disease-free intervals of 10 years or more [57-59]. In fact, a subset of melanomas will have ultra-long dormancy with recurrence greater than 20 years later [60]. Other tumor types, such as lung and pancreatic adenocarcinomas tend to follow a much swifter clinical course in which discovery of the primary tumor and subsequent metastasis is often a temporally contiguous event [61]. While these differences in metastasis patterns may in part reflect differences in detection amongst different cancer types, it has also been proposed that such observations suggest that certain tumor types might gain full metastatic competency earlier in tumor progression [61].

The phenomenon of metastatic dormancy is an intriguing one, and in fact not a new observation. This concept has roots in experiments performed over 50 years ago by Fisher and Fisher [62]. In this study, intraperitoneal injection of small numbers of Walker carcinoma cells did not form visible hepatic tumors when examined five months after tumor cell injection. If however, starting three months after the tumor cell injection, mice were examined every seven days by repeated laparotomy, tumors were visible in 100% of mice by five months [62]. Something about laparotomy influenced growth of macroscopic tumors, suggesting that even though tumors were not visible in control animals, that tumor cells were present, but in a non-proliferative state. Support for the existence of metastatic dormancy in human cancers stems from studies of the growth kinetics of primary and metastatic tumors. The calculated *versus* observed kinetic growth patterns in breast cancer recurrences following mastectomy [63] do not match if one assumes metastatic tumor cells arise late in disease progression, spread, and then continue uninterrupted growth at the metastatic site. Such observations suggest dormancy of disseminated tumor cells may be a real and clinically relevant process in humans.

With this in mind, there has been substantial effort in recent years to understand the mechanisms mediating dormancy of disseminated tumor cells. Tumor cell intrinsic and extrinsic mechanisms have been proposed to explain this process. Metastatic tumor cells, after extravasation, at a metastatic site, find themselves in foreign microenvironments. Tumor cells are known to be very sensitive to changes in microenvironmental conditions, which can lead to alterations of fundamental properties of these cells [64,65]. Experimental evidence in melanoma suggests that induction of apoptosis by Fas/Fas-ligand at metastatic sites is an important negative regulator of metastasis [66]. If individual tumor cells do survive, it is likely that the microenvironment is equipped to either eliminate or prevent growth of these disseminated cells in other ways. Individual disseminated tumor cells purified from the bone marrow of breast cancer patients are generally negative for markers of proliferation [67-69].

One hypothesis explaining dormancy suggests a G0/G1 cell cycle arrest may occur in individual disseminated tumor cells via induction of cyclin-dependent kinase inhibitors [26]. Such cell cycle arrest may be due to interactions (or lack of interactions) with specific components of the microenvironment at the metastatic site [70,71]. It has also been proposed that the immune system may have a role in impeding expansion of dormant tumor cells at metastatic sites [72,73]. Melanoma in particular has long been considered an immunogenic tumor [74], suggesting that immune-mediated destruction of micrometastases may be important. Data from transgenic models of melanoma suggest that cytotoxic CD8+ T cells have antigen specific responses to disseminated tumor cells and may inhibit their proliferation. Depletion of this population of T cells can accelerate progression of metastatic melanoma in mouse models [75-77].

Evidence from human organ transplant literature also implicates the immune system in suppressing the expansion of disseminated tumor cells. The risk of developing primary cutaneous melanomas in immunosuppressed organ transplant recipients is relatively low (3–4× increased risk) compared to other skin cancers such as squamous cell carcinoma (>80× increased risk) [78,79]. However, melanoma is one of the most frequently reported transplant-related malignancies in which metastatic disease develops within the transplanted organ [80]. After transplantation of organs from apparently disease free individuals with a history of melanoma, recipients can develop metastatic melanoma within the transplanted organ. This process is thought to represent reactivation of dormant disseminated tumor cells from the donor. As transplant recipients are immunosuppressed, these scenarios are thought to provide further support for the role of the immune system in suppressing microscopic disseminated disease [80]. Interpretation of the causes of metastatic growth in these rare cases is not entirely conclusive as subclinical graft *versus* host disease in the target tissue may also play a role in increased metastatic growth. In another example, lungs transplanted from a patient with a history of melanoma 32 years prior, gave rise to metastatic melanoma in a recipient within two years of transplantation [81]. Such considerations suggest long-term maintenance of dormancy is possible and enhancement of this process may be therapeutic in patients with melanoma.

Restriction of vascular supply has also been proposed to help maintain metastatic dormancy. In small clusters of disseminated tumor cells, an inability to recruit sufficient vascular supply may restrict growth [26,82]. In mouse models in which angiogenesis is suppressed, lung metastases remain in a non-progressive state, with proliferation balanced by cell death and no net growth of metastatic tumor cell masses [83,84]. Additional evidence from human melanoma biopsies supports this hypothesis, as micrometastases have only half the density of microvessels as do clinically apparent metastases [85]. Kienast and colleagues provide evidence that suppression of angiogenesis may also restrict progression of metastasis in experimental models of melanoma brain metastasis [86]. In separate experiments, Cameron *et al.* have shown that establishment of single disseminated tumor cells occurs equally well in all areas of the lung, but that subsequent growth only occurs in areas either directly adjacent to the vasculature or at the surface of the lungs [87]. Interestingly, dormancy was confirmed in individual cells that did not expand, but persisted and showed neither signs of proliferation nor apoptosis. Such considerations further implicate the importance of interactions between the metastatic cells and the host microenvironment at metastatic sites.

Mechanisms mediating reactivation of disseminated tumor cell growth are very poorly understood. Cell intrinsic factors such as switches in response to TGF-β signaling have been proposed [26]. The HES1 transcription factor, a component of Notch signaling, has been described as an important mediator of the reversibility of quiescence [88] and could theoretically be involved in escape from metastatic dormancy. Certainly, reversal of factors mediating induction of dormancy may mediate escape. For example, changes that allow cells to: overcome cell cycle block, evade immune-mediated growth control, or recruit new blood vessels to support their growth may experience reactivation of growth. Ultimately, mechanisms regulating release from dormancy are likely diverse and highly complex.

## 7. Pre-malignant Dissemination of Melanocytes

The concept of premalignant dissemination posits that cells can spread early in tumor progression and challenges the paradigm that metastasis occurs via late dissemination of rare clones. These “premalignant” cells, without full malignant potential, can be found at sites such as lymph nodes, where they are thought to remain clinically inactive. Pre-malignant dissemination has been invoked to explain metastatic dormancy [89], such that early spread from the primary tumor and late growth at distant sites may help to explain the clinical patterns of metastasis observed in some melanomas. For example, in uveal melanoma, based on calculations of tumor doubling time, it has been proposed that metastases are initiated up to 5 years before identification and treatment of primary lesions [90]. This concept is further supported by the presence of circulating tumor cells in uveal melanoma patients before signs of clinically advanced disease [91,92]. As such, it has been proposed that cells disseminating early may give rise to late-appearing metastases [89].

Substantial data in support of pre-malignant dissemination exists in cutaneous melanoma as well. Four to twelve percent of all patients with metastatic melanoma have no known cutaneous primary lesion [93-95]. This intriguing observation suggests that benign melanocytes may exist at disseminated sites in the body and may be capable of undergoing malignant progression. Further, such progression may be completely unrelated to progression of primary melanomas in these scenarios. In fact, benign melanocytic nevi are often found in lymph nodes. This phenomenon, originally described in the 1930s and observed consistently since then [96-100], is a poorly understood process but certainly consistent with the concept of premalignant dissemination. These “nodal nevi” are seen histologically in 0.33%–7.3% of lymph nodes from non-melanoma patients [101-104]. More sensitive assays, such as quantitative RT-PCR (real-time polymerase chain reaction), suggest that nodal nevi may be even more common than this. Tyrosinase (a pigment production enzyme produced in melanocytes) can be detected in up to 11% of lymph nodes from non-melanoma patients [105,106]. Taube and colleagues recently have identified the BRAFV600E mutation in a substantial subset of nodal nevi [107]. As this same activating mutation is found in the majority of normal cutaneous nevi [20,21] and roughly half of melanomas, it is possible that nodal nevi may arise from the “metastasis” of normal cutaneous nevi. In fact, there is evidence that not only can nevus cells spread to lymph nodes, but they can also enter systemic circulation, where benign melanocytic nevus cells have been detected in human patients [108]. Untransformed cells are capable of extravasation, survival, and even proliferation at distant sites in experimental settings [109]. Benign dissemination has also been observed in other non-malignant conditions such as “benign metastasizing leiomyoma”. In this condition, non-malignant cells derived from growths of smooth muscle cell origin have been reported to metastasize to distant sites, a process thought to represent both lymphatic and hematogenous spread [110]. These considerations suggest that not only does premalignant dissemination occur, but also raise the possibility that it may be a clinically relevant process that could explain some of the clinical patterns observed in melanoma patients.

## 8. Organ-Specificity in Metastasis

Melanoma metastasis involves many steps that are temporally complex. Complicating matters further, Paget's seed-and-soil hypothesis emphasizes the additional spatial complexity of metastasis. Much work in metastasis research has concentrated on the organ-specificity of metastasis and has aimed to identify specific biological mediators of this process. Factors inherent to both tumor cells, as well as the organs to which they metastasize, have a complex interplay that can influence the efficiency with which metastasis occurs to different sites.

Endothelial cells lining the vasculature in different organs differ both structurally as well as at the molecular level. For example, blood vessels in some organs are fenestrated, allowing relatively easy passage of cells from the circulation through large sinusoids. These sinusoidal capillaries are found in the liver, spleen, bone marrow, and lymph nodes. It has been proposed that fenestrated capillaries also allow for easier passage and extravasation of tumor cells [111-113]. While this is almost certainly the case, it is notable that the presence or absence of fenestrated capillaries in target organs does not predict the pattern of metastasis observed in most cancers, including melanoma. At the other end of the spectrum, organs such as the lungs and especially, the brain have structurally sound and continuous endothelial linings that lack pores. Based on this anatomy one might predict that metastasis to these sites might be difficult and thus relatively rare. However, as lung and brain are two of the most common sites of melanoma metastasis [48], the patterns of melanoma metastasis cannot be explained by these considerations alone.

Endothelial cells lining blood vessels have also been shown to express different cell surface receptors and adhesion molecules [114,115]. Based on these differences, it has been proposed that tumor cells expressing cognate receptors or ligands can specifically adhere to the endothelium of target organs [116]. It is quite likely, in fact, that the vasculature of individual organs has a “molecular address” encoded by different endothelial cell surface receptors [117], raising the possibility that interactions with these factors can mediate organ-specific metastasis. In addition to specific adhesion, one must consider extravasation, survival, and subsequent proliferation when studying organ-specific metastasis and that these processes may be governed in different ways in different organs [118,119]. With respect to relative proliferation at metastatic sites, in autopsy series melanoma metastasis is found to occur sub-clinically at many sites, but will only be clinically detectable in a subset of these sites [48]

Chemokine and chemokine receptor interactions have also been implicated in many aspects of tumor cell biology, including metastasis. Chemokines are secreted proteins that can be subdivided into families based on conserved motifs [120]. Based on these motifs, chemokines can interact in specific ways with a diverse group of chemokine receptors [120]. Chemokine/receptor interactions are most well known as mediators of cell migration to specific sites in the body [120]. Chemokines and their receptors are frequently expressed by tumor cells including melanoma [121]. It was proposed in 2003 that the chemokines/chemokine receptors may enable specific interactions between tumor cells and target sites which can influence metastasis [122]. Further, chemokine/receptor interactions can mediate pro-survival signals, which suggests this process may also help explain survival and/or subsequent growth of metastatic tumor cells in particular organs [123].

### 8.1. Metastasis to Lymph Nodes

The first non-contiguous sites to which melanoma cells are thought to spread are lymph nodes [51]. The first lymph node encountered by fluid draining from the cutaneous site where the primary melanoma resides is referred to as the sentinel lymph node. The presence or absence of tumor cells in this lymph node is generally determined in melanoma patients with tumors >1 mm thick in a procedure called a sentinel lymph node biopsy. If the sentinel node is negative histologically, it is likely that other regional nodes are also free of metastasis [124]. The presence of melanoma cells in the lymph node is the single most powerful predictor of recurrence and survival in melanoma patients [125,126], and if it is positive, it is possible that tumor cells have already gained access to the systemic circulation. In fact, removal of the sentinel node or even the entire draining nodal basin does not appear to significantly extend survival in melanoma patients [52,53].

The lymphatic endothelium lacks a well-defined basement membrane, has frequent interendothelial gaps, is inherently leaky, and is as such, thought to provide relatively easy access to tumor cells compared to vascular endothelium [33,127]. Dadras and colleagues have shown that melanomas with lymph node metastasis *versus* those without have a much higher abundance of lymphatic vessels [128]. Increased VEGF-C production, which is involved in lymphangiogenesis [129], may provide a tumor cell intrinsic mechanism promoting increased lymphatic vessel density and thus lymph node metastasis [51]. Other changes to tumor cells, such as changes from more differentiated and non- motile, to less differentiated and more-motile have been implicated in the metastatic process (see below: Epithelial-Mesenchymal Transition), and may have some role in this process.

Metastasis of tumor cells into lymph vessels likely involves reciprocal interactions between tumor cells, immune cells, and the lymph node itself. For example, spread of tumor cells to a lymph node has been shown to result in changes to lymph node biology, resulting in local immunosuppression [130-132]. In fact, elective lymph node dissection in melanoma patients has been shown to alter the pattern (though not the rate) of the first metastatic recurrence [49]. Zhang and colleagues provide intriguing recent evidence that chronic alcohol consumption may even alter lymph node biology in ways that can facilitate lymph node metastasis in melanoma [133].

Physical factors in the lymph node may also affect subsequent spread of tumor cells. Anatomic patterns of lymph flow to a particular lymph node can affect the geographic location of tumor cells within the node [134]. It is certainly possible that the position of tumor cells, perhaps with respect to efferent channels, could play a role in subsequent spread from the lymph node. The load of tumor cells in the lymph node may also be an important factor, as patients with metastatic foci of <0.1 mm have clinical outcomes that are significantly more similar to lymph node negative patients, than to those with lymph node metastases >0.1 mm [135,136].

Chemokine and chemokine receptors have also been proposed to play a role in mediating lymph node metastasis. There is data that CCL21, which is secreted by endothelial cells lining lymphatic channels, may mediate metastasis though interaction with its receptor, CCR7, on melanoma cells [137-139]. Melanoma cells with relatively higher expression of CCR7 are more migratory *in vitro* when exposed to CCL21 [138]. *In vivo* experiments in nude mice also provide support for the importance of this interaction in mediating lymph-node metastasis [139]. Other chemokine/receptor interactions have also been proposed to be relevant to lymph node metastasis. Interactions between CXCL12 and CCL21, chemokines produced by lymph nodes and the CXCR7 and CXCR4 chemokine receptors expressed on melanoma cells, may have similar roles in mediating lymph node metastasis [140]. CXCR3 has also been implicated in lymph node metastasis [141].

### 8.2. Metastasis to Lungs

The lungs and pleura are the most common sites of visceral metastasis in melanoma. One in ten melanoma patients will develop lung metastases at some point in the course of their disease [142]. The lungs are often the first site of visceral metastasis in melanoma [143]. Autopsy series reveal that upwards of 85% of late stage melanoma patients have evidence of lung metastasis [48]. The lungs are common sites of metastasis in many cancers, perhaps in part because blood combined with lymphatic fluid returning from the periphery is first pumped by the right heart through the pulmonary microvasculature. Mouse models of melanoma, including B16, are also most frequently metastatic to the lungs, when metastasis is present [144]. Metastasis to the lungs may be influenced by many factors, including specific adhesive and other molecular interactions. Experimental studies of melanoma metastasis to the lungs have frequently utilized the B16 mouse model of melanoma in which tail vein injection of tumor cells results in lung metastasis. This model has been useful to model the late stages of lung metastasis (*i.e.*, after vascular intravasation), however experimental manipulations that result in reduction or enhancement of lung metastasis are often difficult to interpret in a context of organ-specificity and may represent changes in overall metastatic capability. Nonetheless, there is substantial experimental evidence to suggest that specific molecular interactions may be important in mediating metastasis to the lungs.

Studies in the B16 mouse model of melanoma in the early 1990s showed that B16-F10 melanoma cells (a highly metastatic subline of B16) preferentially adhere to the microvascular endothelium in the lung, but not to control endothelia [145]. Less metastatic B16 variants did not adhere to the lung endothelium suggesting tumor cell intrinsic mechanisms mediate specific adherence to the lung. Also in 1991, Zhu and colleagues, using the B16 model of melanoma, identified Lu-ECAM, an extracellular protein expressed in the lung, as a factor mediating specific adhesion of melanoma cells to the lung [146]. In fact, blocking this adhesion molecule resulted in almost complete elimination of lung metastasis [146]. Years later, it was shown that Lu-ECAM (or CLCA2) is actually a chloride channel expressed predominantly in the lung and is capable of mediating interactions with α6β4-integrin expressed on the tumor cells [147]. This interaction is thought to facilitate metastasis to the lungs. Integrins are not uncommonly identified as mediators such interactions promoting organ-specific metastasis [148,149]. As engagement of integrins and subsequent signaling is also known to mediate cell survival and proliferation [150,151], these interactions probably also provide important survival signals to metastatic cells [152].

Chemokine receptors, including CXCR4, have also been implicated in mediating preferential metastasis to the lung [152-156]. CXCR4 expression in primary melanoma is associated with disease progression [157]. Bartolome *et al.* have recently used a xenograft model of human melanomas to demonstrate the important role of CXCR4 in early phases of melanoma lung colonization [152]. These authors suggest that CXCR4 ligand/CCR12 interaction not only mediates specificity of adhesion, but can also lead to activation of MAPK/ERK and PI3K pathways. Activation of such anti-apoptotic survival pathways is certainly an influential component in the formation of organ-specific metastasis.

### 8.3. Metastasis to Brain

Brain metastasis in all cancers is a notoriously ominous sign as prognosis is particularly poor when this has occurred [158]. Analyses have suggested that 20–54% of melanoma deaths are a result of brain metastases [159-161]. At autopsy, 36–54% of metastatic melanoma patients have brain metastasis, while relatively fewer, 12–20%, have clinically evident metastases during the course of their disease [159-161]. When brain metastases are present, visceral metastases at other sites are also usually present [48]. In the brain, the vasculature is lined by a continuous, non-fenestrated endothelium with tight junctions. This structurally sound endothelial lining is called the blood-brain-barrier and presents a theoretical obstacle for metastasizing tumor cells. The frequency with which brain metastasis is observed in melanoma, however, suggests that melanoma cells are often equipped to cross this barrier. The lack of a lymphatic system is also unique to the brain. Interestingly, the blood brain barrier prevents certain chemotherapy agents from achieving therapeutic doses in the brain and thereby provides an obstacle for treatment. As such, the brain has been referred to as a sanctuary site for melanoma metastasis [162].

The mechanisms regulating metastasis to the brain are relatively poorly understood. Epidemiologic data from melanoma patients suggest that several factors seem to correlate with the development of brain metastasis. Some of these include: male sex (2× risk), primary tumor location on the trunk (72% of brain metastases are thought to originate from primary tumors located above the waist), and primary tumors with superficial spreading histopathology [163,164]. Further, in epidemiologic studies, metastasis to the brain is inversely correlated with metastasis to the liver suggesting these processes may be mediated in different ways [161].

It is clear from autopsy series that metastases to anatomically distinct parts of the brain occur with different frequency. A study by Madajewicz *et al.* suggested that only 4% of melanoma brain metastases occur to lower brain structures [163]. Other studies support this trend and reiterate that most melanoma brain metastases occur to the frontal lobe [48,161]. Metastatic frequency to both hemispheres is equal [48]. Brain metastasis in melanoma shows a preference for the cortex, followed less commonly by sites such as: gray matter nuclei, white matter, leptomeninges, and dura mater. Mouse models of melanoma brain metastasis also show differential metastasis to different parts of the brain. Fidler *et al.* have shown that after injection of different tumor cell lines into the carotid artery of mice, the different lines showed a proclivity for metastasis to different parts of the brain [165]. For example, some lines showed a preference for metastasis to the brain parenchyma, while others showed a proclivity for metastasis to the meninges or ventricles. Follow-up work suggested that TGF-β2 may be important in mediating metastases specifically to the brain parenchyma, but not to the meninges or ventricles [166].

Additional studies in mice have attempted to describe mechanisms modulating metastasis of melanoma to the brain. Some authors have suggested that the expression of the transferrin receptor, through interaction with its ligand transferrin, is important in mediating metastasis of human melanoma cell lines to the brain in mice [167]. Neurotrophins and neurotrophin receptors have also been implicated in the process of brain-specific melanoma metastasis [168]. Specifically, extracellular receptors p75 (the low affinity NGF receptor) and the TrkC receptor tyrosine kinase have been proposed to, via interactions with neurotrophins, NGF and NT-3, mediate brain metastasis in melanoma [169]. In addition to perhaps mediating specificity, there is also evidence that neurotrophins may even help promote colonization of the brain by regulating the production of ECM-degradative enzymes like heparanase [170-172]. Recent studies have also demonstrated the importance of heparanase in the formation of brain metastases by melanoma cells [173]. Additional mediators of brain-specific metastasis in melanoma have been described including chemokines [174], activation of Stat3 signaling [175], and even components of the clotting cascade such as plasmin [176]. The diversity of mechanisms proposed to mediate brain metastasis underscore the complexity of this process.

### 8.4. Metastasis to Other Sites

Hepatic metastases are detected clinically in 10–20% of cutaneous melanoma patients with metastatic disease [48]. Sub-clinical metastases to the liver are much more common, as they are found in 54–77% of melanoma patients at the time of autopsy [56,177,178]. Liver metastases occur relatively late in disease progression, with an average lifespan of only 2–4 months in patients with clinically evident liver metastases [48]. Liver metastases are rarely the first site of disease spread in cutaneous melanoma [48]. Work by Song and colleagues has implicated laminin-1 as a mediator of B16 melanoma cells metastasizing specifically to the liver [179]. In these experiments, cells selected for the ability to adhere to laminin-1 were more efficient in forming liver metastases in mice [179]. Vidal-Vanaclocha and colleagues have implicated interleukins, IL-1β and IL-18 in hepatic metastasis [180]. Mice deficient for IL-1β show an 84-95% reduction in experimental liver metastases. IL-18 is thought to increase expression of VCAM-1 in the hepatic sinusoidal epithelium. Blocking IL-18 with a soluble factor can decrease the adhesion of melanoma cells by inhibiting this mechanism [181]. Laminin-1/VCAM-1 can interact with integrins suggesting again that not only adhesive specificity, but also downstream survival signals are important in determining organ specificity of metastasis.

Bone metastases are relatively uncommon compared to other sites, but are still observed clinically in 11–17% of melanoma patients [48]. In autopsy series they are found in 23–49% patients [56,177,178]. Bone metastases generally occur in patients with late-stage disease, but in a small subset of patients can represent the first site of metastatic recurrence [182,183]. Interestingly, melanoma metastasis to bone generally involves the axial skeleton, most commonly the spine [182,183]. There is recent evidence that targeting TGFβRI can inhibit melanoma metastasis to bone in a xenograft model [184].

Skin and subcutaneous tissue are the most common sites of melanoma metastasis. Cutaneous metastases are thought to be an early external clue that hematogenous spread has occurred [160,185,186]. Chemokines, specifically interactions between CCR10 and CCL27, have been implicated in melanoma metastasis to the skin. CCL27 is a chemokine expressed constitutively in the epidermis by normal keratinocytes [187], and is thought to interact specifically with the chemokine receptor CCR10, which is expressed on melanoma cells [188-190]. Interactions between CCR10 and CCL27 are thought to mediate metastasis to the skin, supported by the observation that neutralizing antibodies to CCL27 can block formation of B16 melanoma in mouse ear skin [191].

Melanomas are also capable of metastasizing to the gastrointestinal (GI) tract, which occur relatively late in melanoma progression. Once GI metastases become clinically evident in melanoma patients, survival averages only 2-4 months [48]. The small intestine is the most frequent site of GI metastasis, though metastases to all parts of the GI tract have been reported [56,177,178]. The chemokine/chemokine receptor pair CCL25/CCR9 has been associated with the ability to metastasize to the small intestine [192]. It has been shown that 86% of melanoma metastases to the small bowel express CCR9, while CCR9 was not significantly expressed in metastases to other organs. CCL25 is produced by the small bowel and thought to mediate specific interaction with CCR9 expressing melanomas [192].

## 9. Other Considerations in Melanoma Metastasis

### 9.1. Epithelial-Mesenchymal Transition

Epithelial-mesenchymal transition (EMT) is a concept that was originally used to describe processes during embryological development in which cells with an epithelial phenotype are converted to cells with a more mesenchymal phenotype. This process is important in developmental processes such as the origin and fate of the neural crest [193]. This concept was subsequently applied to tumor biology to describe the process of tumor progression. In carcinomas, tumors of epithelial origin, EMT posits that loss of differentiated epithelial features such as tight intercellular adhesions, apical-basal polarity, and restricted motility and acquisition of characteristics of undifferentiated, mesenchymal cells such as few intercellular interactions, front-back polarity, and increased cell motility are important in the metastatic process [194]. Functionally, EMT in cancer is thought to be regulated by down-regulation of adhesion molecules like E-cadherin and upregulation of developmentally important transcription factors such as SLUG, SNAIL, TWIST, and ZEB1/2 [194].

EMT in cancer is a controversial topic. Most experimental evidence for EMT is based on *in vitro* studies, leading some authors to call into question the relevance of this process *in vivo* [195,196]. While EMT provides a model for escape from the primary tumor, it does not explain other components of the metastatic cascade such as adhesion, extravasation, survival, and growth at a distant site. More recently, MET (or mesenchymal-epithelial transition), has been suggested to occur at distant sites after an EMT-induced metastasis. Proposed to reconcile the observation that metastases often resemble primary tumors histopathologically [194], there is little direct experimental data to support MET to date [197]. Studies have also indicated that dissemination from primary tumors can be a relatively common events, as shedding of cells from a primary tumor into circulation has been estimated to occur with a frequency of more than a million cells per gram of tumor per day [36]. Further, the presence of tumor cells in the blood does not predict that metastasis will or has occurred [35]. Such additional observations raise the possibility that survival and growth at secondary sites may be more important than acquisition of invasive characteristics and/or escape from the primary site. Moving forward it will be important to take a more global approach to the study of metastasis, that will likely center around new mouse models of melanoma (see Section 10 below: Mouse Models).

Other processes in addition to EMT have been proposed to regulate metastasis at a global level and are reviewed elsewhere [198-200]. In addition to metastasis-promoting factors, metastasis-suppressing factors have also been identified in melanoma. Such suppressive factors include the proteins: KISS1, GPR56, BRMS1, and NEDD9 [201-204].

### 9.2. Organ-Specific Metastasis in the 21st Century

Recent advances in technology have increased the speed and decreased the cost with which high throughput analysis of cancers can be carried out. Several studies have used high throughput approaches and bioinformatic analyses to identify genes associated with metastasis of specific cancers to specific organs [205-208]. Similar studies in melanoma have started to be published, but to date have focused on metastasis in general, rather than to specific organs [209]. Attempts to correlate expression signatures with poor outcome in melanoma have been performed [210-213], but have not reached consensus. Transcripts identified in these studies have had little overlap making interpretation of these data sets difficult [214]. Such discrepancies may have technical aspects, but certainly also reflect the complexity, heterogeneity, and context-specificity of changes in individual melanomas.

In addition to high-throughput expression analysis, next-generation sequencing of cancer genomes and/or exomes is likely to be very informative and identify mutations that drive tumor formation and even metastasis in melanoma. An entire melanoma genome, including annotation of >30,000 somatic mutations, was published earlier this year [215]. In uveal melanoma, exome sequencing has recently identified inactivating mutations to BAP1 in 84% of metastasizing tumors [216]. As the majority of uveal melanomas metastasize to the liver, it will be exciting to see if BAP1 is involved in regulation of metastasis specifically to this site. As additional cutaneous melanoma genomes become available, a more comprehensive view of somatic mutations that drive melanoma will be available. Such knowledge will allow for study of organ-specific metastasis in relevant genetic contexts. For example, it is possible that mechanisms regulating metastasis in certain genetic contexts may not regulate metastasis in other genetic contexts.

### 9.3. Genetics of Melanoma and Metastasis

Despite ongoing efforts to characterize additional genetic mutations mediating melanoma formation and progression, quite a bit is already known [217]. BRAF, a mitogenic Ser/Thr kinase in the MAPK/ERK pathway, has an activating mutation, V600E, in at least 50% of melanomas [218,219] and is thought to be a central oncogenic driver in melanoma. The effect of BRAFV600E mutational status on melanoma metastasis is still somewhat unclear, though some data do exist. BRAF-mutant tumors have been reported to have a worse prognosis than, for example, NRAS mutants [220], another oncogenic mutation that is thought to drive melanoma formation in a smaller subset of tumors. Though, the association of BRAF-mutation with poor outcome compared to NRAS-mutants was not replicated in other studies [221,222]. Some preliminary evidence suggests that inhibition of targets downstream of mutant BRAF in melanoma can inhibit lung metastasis [223]. Evidence from thyroid cancer, in which the BRAF V600E mutation is also common, suggests that this mutation can increase invasiveness [224,225]. However, a recent study in an orthotopic mouse model of melanoma has suggested that RAS/RAF mutational status does not have a role in determining metastasis [226]. The discrepancies in these early studies suggest the implications of the BRAFV600E mutational status with respect to survival and metastasis in melanoma are likely complex.

The PI3K/AKT pathway regulates cell survival, growth, and proliferation. This pathway is often dysregulated in melanoma through various mechanisms including inactivation of PTEN phosphatase, which is mutated in 5-20% of primary melanomas [227-229]. Protein expression of PTEN is lost through other mechanisms including epigenetic changes in a larger subset of melanomas [230,231]. With respect to metastasis, it has been shown that adding PTEN back to PTEN-deficient cells can diminish metastasis [232]. Pten loss has also been shown to enhance melanoma metastasis in mice [233,234]. It is currently unclear if signaling through this pathway is capable of mediating metastasis to particular organs rather than influencing metastasis more generally. As a comprehensive understanding of the driving events in melanoma is developed, more powerful and relevant analyses of the mediators of the metastatic cascade will be possible.

### 9.4. Additional Considerations

Cancer stem cells, or tumor-initiating cells, are a subpopulation of cells within a tumor that are thought to be relatively more tumorigenic than other tumor cells, relatively resistant to chemotherapy, and to mediate disease recurrence after treatment [235,236]. The cancer stem cell model, first developed in leukemias, has since been applied to solid tumors including melanoma. Much effort has gone into identifying and characterizing this population of cells in melanoma [237-242], but consensus has yet to be reached. The role of tumor-initiating cells in melanoma metastasis is relatively uncharacterized. Further, similarities and differences in the processes mediating tumor-initiating cell biology and metastatic dormancy are also unknown. It has even been proposed that mechanisms regulating EMT may relate to tumor-initiating cell biology [243]. It will be important to clarify the relationship of these processes to both each other as well as to tumor heterogeneity more generally. Better understanding of mechanisms regulating these processes in melanoma will aid in the study of their relationship to metastasis, dormancy, and tumor recurrence.

MicroRNAs (miRNAs) are short RNA molecules encoded within the genome that are capable of negative post-transcriptional regulation of target mRNAs. miRNAs are known to be dysregulated in cancers including melanoma [244] and have been shown to be functional mediators of metastasis [245,246]. miRNAs are thought to be capable of regulating complex biological processes through their ability to functionally repress many transcripts that may have a related function. As such, miRNAs may have important roles in influencing metastasis, dormancy, and reactivation. The dysregulation of several miRNAs has already been proposed to have functionally important consequences in melanoma [247-249]. Large scale characterization of miRNAs in melanoma continues and will likely be pivotal in more completely understanding the process of metastasis.

Although not addressed in detail within this review, the immune system (and more broadly the tumor microenvironment) has been shown to play an important role in melanoma formation, progression, and metastasis. The capacities in which the immune system can interact with melanomas are complex and can be different in different circumstances. Some authors have implicated components of the immune system such as macrophages as having a primarily pro-tumor and pro-metastasis function [250-253], while other authors have identified components of the immune system that can negatively regulate melanoma and melanoma metastasis [254]. Enhancement of immune-mediated tumor rejection is a central means by which promising new melanoma therapies function [255]. Other components of the tumor microenvironment can play roles in melanoma formation and metastasis [256]. The context of a “pre-metastatic niche” and its importance in influencing tumor metastasis is another microenvironmental consideration undergoing intensive investigation [257-259].

## 10. Mouse Models

In recent years, improvements in mouse models based on melanocyte-specific, conditional Cre-lox recombination technology now allow for reproducible formation of spatially restricted melanomas in immune-competent mice [233]. In these models melanoma-relevant genetic changes such as Pten loss and Braf activation drive transformation of normal mouse melanocytes into melanoma leaving surrounding tissue unaltered, thus recapitulating complex host-tumor interactions that occur through tumor development. Other, more highly metastatic models have been generated based on additional genetic changes and will allow for the evaluation of the individual steps in the metastatic cascade [260]. Fluorescent reporters in these endogenous mouse models of melanoma will allow for tracking of individual tumor cells and purification of these cells at different steps of the metastatic process. For example, the temporal kinetics between metastasis to lymph node, presence of circulating tumor cells, and metastases at distant sites can be easily characterized utilizing these technologies. This next generation of mouse models will not only be important discovery tools, but will also be a means by which to test additional hypotheses. For example, the relevance of EMT in melanoma could be directly tested by either inactivating E-cadherin or over-expressing particular transcription factors in these models. As more and more strains of mice become readily available, such models will provide a platform for testing the roles of other potentially modulating changes with respect to melanoma formation and progression. More broadly, careful study of organ specificity, premalignant dissemination, and metastatic dormancy are now possible in these models, which will be central to advancing our understanding of basic processes in melanoma metastasis.

## 11. Conclusions

The process of melanoma metastasis is quite complex. Early models of melanoma formation and metastasis have been useful in advancing our understanding of melanoma progression. However, as we learn more about the heterogeneity of the metastatic process in melanoma it becomes clear that these models must be amended to address additional complexities of metastasis. Concepts such as metastatic dormancy, premalignant dissemination, and organ-specific metastasis should be incorporated into newer models of metastasis. Moving forward, it will be important to better understand each of the component steps of metastasis at a mechanistic level and to develop clear descriptions and definitions of these processes so that they may be related to other entities in melanoma research, such as tumor heterogeneity and tumor-initiating cells. Clinical and *in vivo* observations should be kept in mind when interpreting the applicability of *in vitro* experimental findings. For example, mediators of cell motility *in vitro* probably do not always equate with increases in metastasis *in vivo*. Furthermore, entry into circulation through increases in cell motility is only one step in a complex process, other factors such as those mediating survival of tumor cells in circulation, extravasation at distant sites, survival, dormancy, and subsequent proliferation are important and cannot be ignored.

Individual melanomas are driven by diverse genetic and epigenetic alterations. It may be difficult to determine a universal set of the factors mediating melanoma metastasis, as changes that mediate metastasis in one melanoma may not mediate this process in another. Also, factors mediating survival and proliferation of a metastatic tumor cells in the liver, for example, may be vastly different than factors mediating this process in the brain. Moreover, treating metastases to these different organs may require different strategies that will also be confounded by different driving mutations in individual tumors. Ultimately, tumors develop and metastasize in the experimentally intractable setting of individual patients, in which comorbid conditions such as metabolic disorders and chronic inflammatory states also influence disease progression in ways that are very complex and only beginning to be understood.

Finding core mediators of different processes in metastasis is a formidable challenge, but will provide opportunities for developing new treatment strategies. Targeting these processes with new therapeutic agents and implementing them in appropriate clinical settings will present additional challenges. Metastasis research has progressed immensely since the time the seed-and-soil hypothesis was originally proposed, though there are additional subtleties to the process that we are just beginning to understand. As human melanoma is more broadly characterized through extensive sequencing of melanoma genomes, a comprehensive view of mutations driving melanoma formation and progression will be determined in the next few years. Given the rapid progress of technology in other areas such as proteomics, imaging, mouse modeling, and structure-based drug design, description and exploitation of newly discovered changes can occur rapidly, providing unprecedented opportunities for both understanding and treating melanoma in the years to come.
